# Female signalling to male song in the domestic canary, *Serinus canaria*

**DOI:** 10.1098/rsos.140196

**Published:** 2015-01-28

**Authors:** Mathieu Amy, Pauline Salvin, Marc Naguib, Gerard Leboucher

**Affiliations:** 1 Laboratoire Ethologie Cognition Développement, EA 3456, Université Paris Ouest – Nanterre La Défense, 200 Avenue de la République, Nanterre 92000, France; 2 Behavioural Ecology Group, Department of Animal Sciences, Wageningen University, Wageningen, The Netherlands

**Keywords:** sexual selection, female-specific signals, male–female vocal interaction, copulation solicitation, songbird

## Abstract

Most studies on sexual selection focus on male characteristics such as male song in songbirds. Yet female vocalizations in songbirds are growing in interest among behavioural and evolutionary biologists because these vocalizations can reveal the female's preferences for male traits and may affect male display. This study was designed to test whether male song performance influences the different female signals in the domestic canary (*Serinus canaria*). Female canaries were exposed to three types of song performance, differing in the repetition rate of sexy syllables. This experiment demonstrates that female birds are engaged in multimodal communication during sexual interaction. The results support the copulation solicitation hypothesis for female-specific trills: these trills were positively correlated and had a similar pattern to the copulation solicitation displays; responses were higher to the songs with higher performance and responses decreased with the repetition of the stimulation. Also, we observed a sensitization effect with the repetition of the song of the highest performance for the simple calls. Simple trills and other calls were more frequent during the broadcast of canary songs compared with the heterospecific control songs. The differential use of female signals in response to different song performance reveals a highly differentiated female signalling system which is discussed in light of the role of female traits to understand sexual selection in a broader perspective.

## 2. Introduction

Since Darwin [1], the development of the theory of sexual selection mainly focused on male secondary sexual characteristics. Extravagant traits such as colourful plumages, courtships or vocalizations of males have been the object of a myriad studies [2]. Yet studies on animal reproduction are often male-biased, and this bias could lead to extrapolations or generalizations that could be misleading [3]. Females also express secondary sexual characteristics [4,5] and it is unlikely that it represents an artefact of the selection of male secondary sexual characteristics [6]. Females in many species indeed compete for access to a reproduction territory, to other resources, or to a male [7].

Most studies on animal communication in songbirds have mainly focused on male song [5,8], which has been shown to underlie sexual selection and to which one usually attributes two main functions: mate attraction/stimulation and territory defence against rivals [9,10]. However, there is a new interest in the vocal signals of female songbirds. Duet and solo songs of female birds are of growing interest among behavioural and evolutionary biologists [11–15]. Female song has similar functions to male song [11,14,16] and was probably the ancestral state in songbirds [5,17,18].

Other studies have focused on female calls in birds. Functions of calls in birds are diverse, such as to maintain social/pair bonds, to beg for food, to threat and to alarm [19,20]. Male and female call repertoires overlap in part or entirely [13] but it is likely that, in all species, both sexes have sex-specific calls, only produced by one sex, or sex-typical calls, mainly produced by one sex [21]. Several ultimate hypotheses can be used to explain female sex-specific or female sex-typical calls in birds, such as the self-stimulation hypothesis [22], the copulation solicitation hypothesis [23], the direct and indirect mate-sampling hypotheses [24,25] and the female–female competition hypothesis [12].

Birds often use multimodal signalling by combining movements with vocalizations in various contexts such as sexual interactions (e.g. [26]), aggressive interactions (e.g. [27]) and begging for food (e.g. [28]). So far, the understanding of female multimodal signals and their function is still poor.

**Figure 1.**
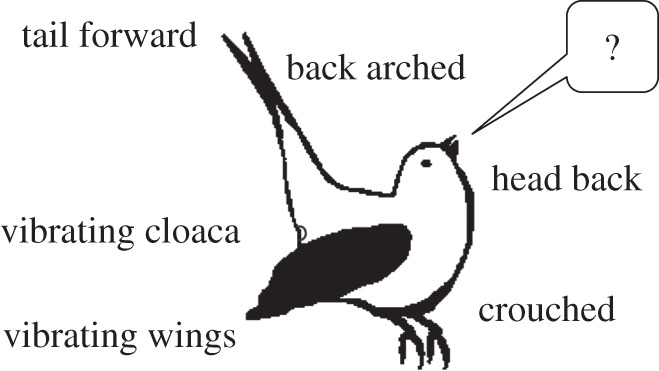
Schematic representation of copulation solicitation display in female canary.

A well-known visual signal of female songbirds is the copulation solicitation display (CSD). During a CSD (figure 1), the female crouches, arches her back and simultaneously brings her tail forward and her head back, moves her wings away from her body and vibrates the wings and the cloaca [29]. The counting of the CSDs elicited by females for different songs is one of the most widely used methods to assess female preferences within songbirds [30,31]. To assess female preferences, it is important to find reliable and informative measures [32] and female preferences should *a priori* predict female mate choice. Since CSDs precede copulation and are a signal emitted by the female towards the male, inviting him to copulate, CSD displays towards playback of song can be used as indicator of female song preference. For instance, female canaries (*Serinus canaria*) perform more CSDs for particular song phrases, named ‘A-phrases’ or ‘sexy phrases’ [33,34]. Female canaries also perform more CSD for male songs maximizing both the syllable rate and the frequency bandwidth [35]; there is a general trade-off relationship between the frequency bandwidth and the syllable repetition rate within the song [35–37].

This study aims to investigate whether female birds use multimodal signals to solicit copulation. Female canaries emitted more simple calls (SCs) when they heard a song containing ‘A-phrases’ (also known as ‘sexy phrases’) when compared with songs without ‘A-phrases’ [38]; as these results are similar to the CSD assay, the authors suggested that female canaries can also show their preferences by calling [38]. In a further study [39], they showed that SCs were also used in maintenance of flock cohesion. In addition, Mulligan & Olsen [40] investigated the ‘communicatory value’ of the different calls in the domestic canary and they described the SCs rather as ‘anxiety calls’ [40], p. 179. Moreover, they described one or two types of repeated calls (or trills), labelled as ‘mating call’, mainly produced by females and ‘often leading to either courtship feeding or copulation’ [40, p. 180]. Yet, at the time, the authors did not provide quantitative evidence of the potential mating or courtship function of these female-specific trills (FSTs); these specific trills were often heard in experiments on female canaries during the broadcast of male songs and were never heard outside the broadcast period (E. Vallet 2011, personal communication).

Here, we tested whether male song performance influenced female signals in the domestic canary. During a reproductive cycle, females were exposed daily to three types of songs: two songs of a male domestic canary with different song performances (‘sexy phrases’ with different repetition rate) and one heterospecific song (zebra finch, *Taeniopygia guttata*, song) as a control; each was presented three times in a row. We noted the different signals emitted by females: CSDs, SCs, FSTs, simple trills (STs) and other calls (OCs). We hypothesized that female signals would vary with male song performances and, more specifically, that females would perform more CSDs and more SCs to songs of higher performances, as has been shown in previous studies [35,38]. We predicted the same results for the FST described by Mullingan & Olsen [40] as a ‘mating call’. Also, we hypothesized that females would respond with more STs and OCs to conspecific songs rather than heterospecific song but that these vocalizations would not change with male song performance [38]. We secondly hypothesized that a habituation effect would be observed with the repetition of the stimulation for CSD [41], for SC and FST but not for the ST and OC. Finally, we also predicted that CSD, SC and FST would be positively correlated.

## 3. Material and methods

### 3.1 Subject and breeding condition

We used 16 sexually mature domestic canary females (ages: 3–5 years), hatched and reared in our laboratory and with previous experience of reproduction. Before the experiment, all females were maintained in social groups in aviaries (118×50×50 cm), on a short-day photoperiod (8 L : 16 D) for a minimum of four months. During the experiment, all females were housed in individual cages (38×22×26 cm) and placed individually in sound-proof chambers (68×51×51 cm) on a long-day photoperiod (16 L : 8 D), stimulating their reproductive behaviour. Females were provided with water and seeds daily ad libitum and with breeding food twice a week. Females' cages were equipped with two perches, nest bowls and nesting material.

### 3.2 Experimental songs

**Figure 2.**
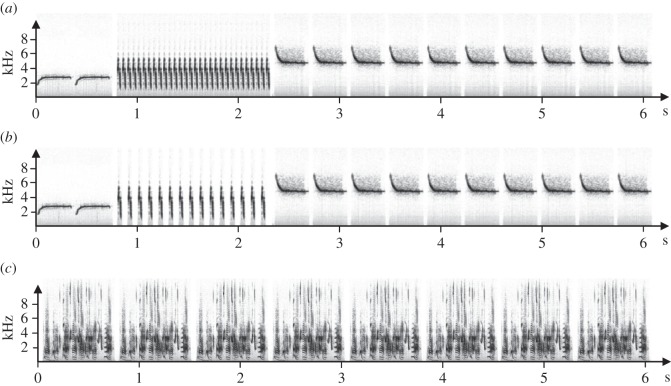
Sonograms of the different types of broadcasted songs. (*a*) Sonogram of a canary song containing a very attractive A-phrase (A20); (*b*) sonogram of a canary song containing a moderately attractive A-phrase (A10) and (*c*) sonogram of a zebra finch song (ZF).

Thirty-two canary songs were constructed from 16 songs of male canaries recorded during the preceding years in our laboratory. The digitalization and construction of the song files was done with Avisoft SASLab-Pro software (Raimund Specht, Berlin, Germany) with a sample frequency of 22 050 Hz. All songs lasted for 6 s and were structured as follows: two introductory notes (0.75 s), then one ‘A-phrase’ (1.50 s) and six conclusive notes (3.75 s). The A-phrases elicit strong sexual responses by female canaries [33], and female canaries show higher sexual responses when the repetition rate of the syllable in the A-phrase increases [35]. We generated 16 songs containing very attractive A-phrases with two distinctive notes repeated at a rhythm of 20 syllables s^−1^. Each of these 16 songs was constructed from 16 different A-phrases. Then we used these songs to generate 16 songs containing a moderately attractive A-phrase with two distinctive notes repeated at a rhythm of 10 syllables s^−1^. As a control, we used three different natural songs of zebra finches to generate three different 6-s songs from the repetition of the natural songs. Thus, we had three types of song stimuli: 16 canary songs containing a very attractive A-phrase (A20), 16 canary songs containing a moderately attractive A-phrase (A10) and three zebra finch songs (ZF) (figure 2).

All songs were adjusted to the same amplitude and were passed through a band pass filter (0.8–8 KHz). We then constructed song bouts. A song bout was composed of three identical 6-s songs followed by 14 s of silence. Finally, we constructed song sequences. A song sequence was composed of three identical song bouts. Therefore, one song sequence lasted for 1 min. Each female was randomly assigned to one A20 song and one A10 song, and each of the three zebra finch songs was randomly assigned to a female.

### 3.3 Trials

Song sequences were broadcasted with a natural sound pressure level (i.e. approx. 65 dB measured at a distance of 30 cm from the loudspeaker) by one DELL loudspeaker (HK206) connected to a digital audio player able to play WAVE files (Philips Gogear). Trials were performed from the first day of the long-day photoperiod and stopped the day the female laid her last egg (34 ± 3 (mean ± s.e.) days of trial from the onset of the long-day photoperiod). Females were tested once daily every morning. The different types of song sequences were presented one after the other, with 2 min of silence between them. The order in which the types of songs were presented was balanced.

During each trial, we recorded female vocalizations with a microphone (Anchor MIC-50) connected to a digital recorder (Marantz PMD 670) with a sample frequency of 44 100 Hz. We also recorded the female behaviour with a digital camera (Casio Exilim) placed outside the sound-proof chamber.

**Figure 3.**
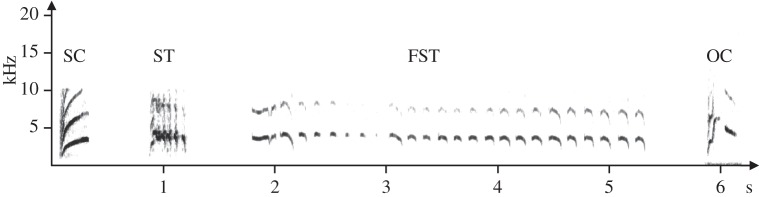
Sonogram of the different call types. SC, simple call; ST, simple trill; FST, female-specific trill; OC, other call.

We measured different female signals: (i) the number of CSDs. Additionally, we also noted the number of different calls emitted by females and distinguished four types of calls (figure 3) using sound spectrograms (Avisoft SASLab-Pro software; Raimund Specht, Berlin, Germany): (ii) SC, (iii) ST, (iv) FST, and (v) OC. During CSD, the female crouches, arches her back and simultaneously brings her tail forward and her head back, moves the wings away from the body and vibrates them and the cloaca [29]. SCs are the contact calls consisting of a single note. STs are ‘repeated calls’ [40] consisting of more than one note, emitted in a rhythmic sequence with a constant sound pressure level. FSTs are all calls consisting of more than one note but emitted in an arrhythmic sequence [40] with variation in the sound pressure level. STs also generally consist of few notes, whereas there are many variations in the number of notes within FSTs (M.A. 2011, personal observation). OCs are calls that could not be categorized in the three other types of calls. To investigate the temporal use of the different vocalizations by females, we used sound spectrograms to measure (vi) the latencies of the different calls from the onset of the playback. Finally, we also measured (vii) the duration of the FSTs to investigate the effect of song performance on females' signals duration.

### 3.4 Statistical analyses

To assess the relationships between the different signals emitted by females during the different trials, we used the total number of calls of each type given in the different trials to perform Spearman correlation tests.

To test whether song performances, the repetition of the stimulation (three times in a row) and their interaction influenced the occurrence of each signal, we used general linear mixed models (GLMMs) using library ‘lme4’ of the R software (v. 2.11.1). To obtain an orthogonal design, for each female and for each signal, we used the sums in each stimulation repetition according to the different song performances. GLMMs allowed us to construct a model with both fixed effects and random effects [42], and to specify data distribution (here a Poisson distribution); we specified the focal female as the random factor (to control for repeated measures) and the song type, the repetition of the stimulation and their interaction as the fixed factors. We then used a frequentist null-hypothesis test approach [43] to test for the fixed effects and their interaction. For each variable, we first tested the model with the interaction against the model without the interaction. If the interaction was not significant, we removed it from the model and then tested the new model against the minimal model. Finally, we performed Tukey *post hoc* tests when appropriate, using library ‘multcomp’ of the R software (v. 2.11.1).

To investigate the temporal use of the different vocalizations by females, we used mean latencies of each female for each type of call. Since all females did not utter all call types, we used GLMM using library ‘lme4’ of the R software (v. 2.11.1). This method allowed us to specify the focal female as the random factor to control for both repeated measures and that all females did not utter all call types. The call type was specified as the fixed factor. We then tested this model against the minimal model and Tukey *post hoc* tests were performed, using library ‘multcomp’ of the R software (v. 2.11.1).

To test whether song performance, the repetition of the stimulation (three times in a row) and their interaction influenced the duration of the FSTs, for each female and for each signal, we used the sums of FSTs' length in each stimulation repetition according to the different song performances. Since not all females emitted FSTs, we used GLMM using library ‘lme4’ of the R software (v. 2.11.1). This method allowed us to specify the focal female as the random factor to control for both repeated measures and that not all females emitted FSTs. The song type, the repetition of the stimulation and their interaction were specified as the fixed factors. We first tested the model with the interaction against the model without the interaction. If the interaction was not significant, we removed it from the model and then tested the new model against the minimal model. Finally, we performed Tukey *post hoc* tests when appropriate, using library ‘multcomp’ of the R software (v. 2.11.1).

## 4. Results

### 4.1 Relationships between copulation solicitation displays and female vocalizations

There was no correlation among female signals (all *p*>0.10) except that FSTs were significantly positively correlated with CSDs (*r*=0.69, *p*=0.003, *n*=16, Spearman's rank-order correlation, table 1).

**Table 1.** Spearman's rank-order correlations of females' signals. The significant correlation (*p* = 0.003) is given in bold. CSD, copulation solicitation display; FST, female-specific trill; OC, other call; SC, simple call; ST, simple trill.

	FST	OC	ST	SC
CSD	**0.69**	0.06	0.33	−0.03
FST		−0.28	0.25	0.32
OC			−0.42	−0.12
ST				−0.07

### 4.2 Effects of song performance and of stimulus repetition on female signals

**Figure 4.**
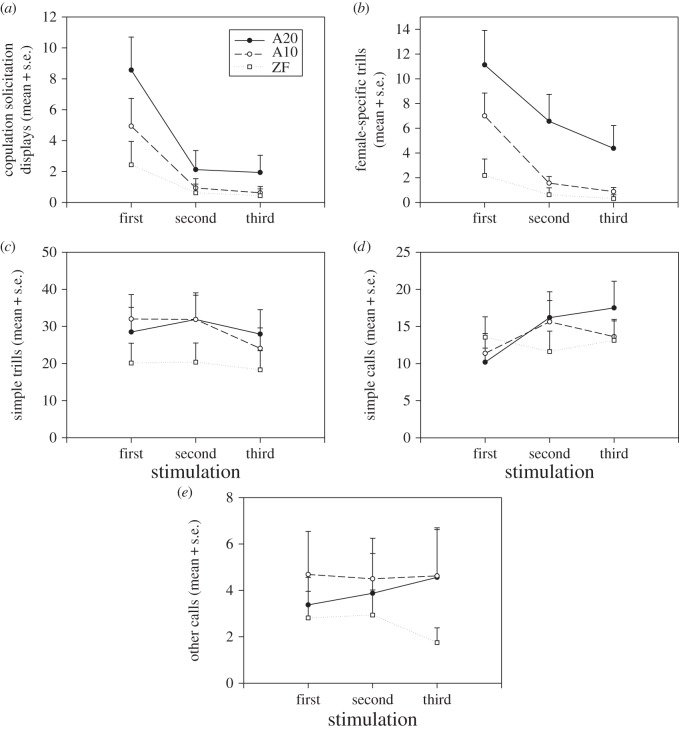
Females' signals in response to different male songs, according to the repetition of the stimulation. (*a*) CSDs, (*b*) FSTs, (*c*) STs, (*d*) SCs and (*e*) OCs. Filled circles (A20), canary song containing a very attractive ‘A-phrase’ (syllable rate: 20 s^−1^); open circles (A10), canary song containing a moderately attractive ‘A-phrase’ (syllable rate: 10 s^−1^); open squares (ZF), zebra finch song. Mean + s.e. are presented.

The occurrence of the CSDs was affected by song performance (GLMM; *χ*^2^=91.3; *p*<0.0001) and the repetition of the stimulation (GLMM; *χ*^2^=208.7; *p*<0.0001) but there was no significant interaction between song performance and the repetition of the stimulation with the occurrence of the CSDs (GLMM; *χ*^2^=2.8; *p*=0.592). Tukey *post hoc* tests show that CSDs were significantly more common for songs of higher performance, and we observed a significant decrease of the CSDs with the two first repetitions of the stimulation independently of song performance, but no decrease was observed for the third repetition of the stimulation (table 2 and figure 4*a*); *n*=16 for each test.

**Table 2.** Pairwise multiple comparisons of the occurrences for each signal. CSD, copulation solicitation display; FST, female-specific trill; SC, simple call; ST, simple trill; OC, other call. Tukey *post hoc* tests were performed. *n*=16 for each test. Significant values are given in bold.

pairwise multiple comparisons	CSD		FST		ST		SC		OC	
	*q*	*p*	*q*	*p*	*q*	*p*	*q*	*p*	*q*	*p*
song										
A20 versus A10	−5.50	<**0.001**	—	—	−0.11	0.993	—	—	1.58	0.254
A20 versus ZF	−8.49	<**0.001**	—	—	−9.66	<**0.001**	—	—	−3.89	<**0.001**
A10 versus ZF	−3.73	<**0.001**	—	—	−9.55	<**0.001**	—	—	−5.38	<**0.001**
stimulation										
1 versus 2	−10.12	<**0.001**	—	—	1.09	0.520	—	—	—	—
1 versus 3	−10.61	<**0.001**	—	—	−3.36	**0.002**	—	—	—	—
2 versus 3	−1.06	0.532	—	—	−4.44	<**0.001**	—	—	—	—
stimulation within song A20										
1 versus 2	—	—	−4.29	<**0.001**	—	—	4.63	<**0.001**	—	—
1 versus 3	—	—	−6.61	<**0.001**	—	—	5.49	<**0.001**	—	—
2 versus 3	—	—	−2.63	0.147	—	—	0.90	0.993	—	—
stimulation within song A10										
1 versus 2	—	—	−6.78	<**0.001**	—	—	3.26	**0.031**	—	—
1 versus 3	—	—	−7.33	<**0.001**	—	—	1.80	0.682	—	—
2 versus 3	—	—	−1.74	0.682	—	—	−1.48	0.865	—	—
stimulation within song ZF										
1 versus 2	—	—	−3.49	**0.011**	—	—	−1.54	0.834	—	—
1 versus 3	—	—	−4.07	**0.001**	—	—	−0.34	1.000	—	—
2 versus 3	—	—	−1.27	0.927	—	—	1.21	0.955	—	—
song within stimulation 1										
A20 versus A10	—	—	−3.84	**0.003**	—	—	1.02	0.984	—	—
A20 versus ZF	—	—	−8.79	<**0.001**	—	—	2.76	0.126	—	—
A10 versus ZF	—	—	−6.00	<**0.001**	—	—	1.75	0.714	—	—
song within stimulation 2										
A20 versus A10	—	—	−6.45	<**0.001**	—	—	−0.40	1.000	—	—
A20 versus ZF	—	—	−7.10	<**0.001**	—	—	−3.45	**0.016**	—	—
A10 versus ZF	—	—	−2.45	0.221	—	—	−3.05	0.057	—	—
song within stimulation 3										
A20 versus A10	—	—	−5.50	<**0.001**	—	—	−2.77	0.123	—	—
A20 versus ZF	—	—	−5.70	<**0.001**	—	—	−3.15	**0.043**	—	—
A10 versus ZF	—	—	−1.98	0.512	—	—	−0.39	1.000	—	—

There was a significant interaction between song performance and the repetition of the stimulation for the occurrence of the FST (GLMM; *χ*^2^=28.8; *p*<0.0001). Tukey *post hoc* tests show that females uttered significantly more FSTs for songs of higher performance, and use of FSTs decreased significantly with the repetition of the stimulation (i.e. an habituation affect) but this decrease was less pronounced for the song of the highest performance A20 (table 2 and figure 4*b*); *n*=16 for each test.

There was no significant interaction between song performance and the repetition of the stimulation on the duration of the FST (GLMM; *χ*^2^=3.2; *p*=0.54). The duration of the FST was not affected by song performance (GLMM; *χ*^2^=4.61; *p*=0.1) but was significantly affected by the repetition of the stimulation (GLMM; *χ*^2^=18.3; *p*<0.0002). Tukey *post hoc* tests show that the duration of FST decreased significantly with the repetition of the stimulation; the duration of the FST was significantly longer for the first stimulation compared with the second stimulation (*q*=−3.8; *p*<0.001) and the third stimulation (*q*=−4; *p*<0.001). There was no significant difference in the duration of the FST uttered during the second stimulation compared with the third stimulation (*q*=−0.5; *p*=0.872).

Also the number of STs was significantly affected by song performance (GLMM; *χ*^2^=122.9; *p*<0.0001) and by the repetition of the stimulation (GLMM; *χ*^2^=21.4; *p*<0.0001), but there was no significant interaction between song performance and the repetition of the stimulation on the number of the STs (GLMM; *χ*^2^=8.9; *p*=0.063). Tukey *post hoc* tests show that females responded significantly less with STs for the heterospecific zebra finch control song compared with the two male canary songs but performance of the two different canary song versions did not affect the occurrence of STs. Moreover, females emitted significantly fewer STs at the third repetition of the stimulation compared with the first and the second stimulation (table 2 and figure 4*c*); *n*=16 for each test.

The occurrence of the OC was significantly affected by song performance (GLMM; *χ*^2^=31.6; *p*<0.0001) but not by the repetition of the stimulation (GLMM; *χ*^2^=0.2; *p*=0.922). There was no significant interaction between song performances and the repetition of the stimulation and the number of the OCs (GLMM; *χ*^2^=8.6; *p*=0.073). Tukey *post hoc* tests show that females uttered significantly fewer OCs for the heterospecific zebra finch control song compared with the two male canary songs, but performance of male canaries' songs did not affect the occurrence of OC (table 2 and figure 4*e*); *n*=16 for each test.

There is a significant interaction between song performances and the repetition of the stimulation for the occurrence of SCs (GLMM; *χ*^2^=28.1; *p*<0.0001). Tukey *post hoc* tests show that there is a significant increase of the number of SCs with each repetition of the stimulation but only for the song of highest performances, A20. For the song of moderate performances, A10, this increase was significant only between the first and the second stimulation but not between the second and the third stimulation. Finally, there is no such increase for the heterospecific control song (table 2 and figure 4*d*); *n*=16 for each test.

### 4.3 Latency of the different vocalizations

**Figure 5.**
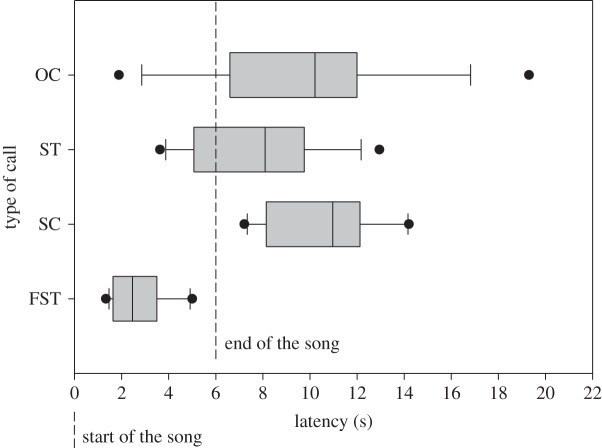
Latencies of the different vocalizations relative to the start of the broadcast of the song. Median, upper quartile, lower quartile and outliers are presented. The start and the end of the song are presented for information. FST, female-specific trill; OC, other call; SC, simple call; ST, simple trill.

The different call types were uttered with a different temporal pattern relative to the beginning of the song (GLMM; *χ*^2^=49.35, *p*<0.0001). *Post hoc* statistical analyses show that FSTs are uttered with significantly shorter latencies compared with all other types of vocalization (table 3 and figure 5). Also, STs are uttered with significant shorter latencies than SCs (table 3 and figure 5).

**Table 3.** Pairwise multiple comparisons of the latencies to emit the different signals. FST, female-specific trill; OC, other call; SC, simple call; ST, simple trill. Tukey *post hoc* tests were performed. Significant values are given in bold.

pairwise multiple comparisons	*Z*	*p*
OC versus FST	6.65	<**0.001**
SC versus FST	7.80	<**0.001**
ST versus FST	5.08	<**0.001**
SC versus OC	0.76	0.873
ST versus OC	−1.86	0.244
ST versus SC	−2.77	**0.029**

## 5. Discussion

The experiments provide quantitative evidence of the relationships between different signals used by female birds during inter-sexual communication. Utterances of the different signals were not correlated with each other, except for only two signals, suggesting that most signals indeed have different functions. The experiments show that FSTs were significantly positively correlated with CSDs, whereas neither SCs, STs nor OCs were correlated with CSDs, nor with FSTs, nor to each other. Moreover, the experiments reveal that the use of the different female signals was differentially affected by song performance and by the repetition of the stimulation. First, the two correlated signals (CSDs and FSTs) were expressed with similar patterns (but see below for some differences): CSDs and FSTs were always more common for songs of higher performance and there was a habituation effect with the repetition of the stimulation. The duration of FST was only affected by the repetition of the stimulation but not by song performance. Then, there was a sensitization effect with the repetition of the song of highest performance for the SC. Finally, STs and OCs were less common for the heterospecific song.

The pattern of results for the CSD is in line with the pattern found in previous studies (e.g. [35,41]) and the use of FST was very similar to the CSD pattern. The habituation across repeated playbacks depended on song performance for the FST, but this habituation effect was well less pronounced for the song of the highest performances A20. Moreover, CSDs and FSTs frequently were emitted independently of one another. Therefore, it is unlikely that the FST is a by-product of wing vibrations or cloaca vibrations observed in CSD. Mechanistically, FST and CSD may well be associated with the same hormonal mechanisms, as plasmatic oestradiol concentrations play a crucial role in triggering CSD in female canaries (see review in [31]). A threshold level of oestradiol is probably needed to activate the neural circuitry mediating the CSD [31] and we assume that oestradiol may also be involved in the neural circuitry mediating the FST; endocrine mechanisms of female-typical or female-specific calling are still widely unknown [21].

Our results also reveal an unexpected significant interaction between song performances and the repetition of the stimulation for the SC. Contrary to our expectations, we observed a sensitization effect with the repetition of the song of highest performance (A20) but this sensitization effect was inexistent for the control song and less pronounced for the song with moderate performances (A10). These results do not support a previous study suggesting that female canaries can also show their preferences with SC [38]; this study clearly suggests that SC and CSD lead to similar patterns of responses. However, Nagle and his collaborators [38] used song sequences consisting of 10 repetitions of the same song, whereas our song sequences consisted of three repetitions of the same song and we found a sensitization effect with the repetition of the stimulation for the SC uttered during the broadcast of the song with the highest performance. This sensitization effect may explain why Nagle and collaborators [38] found that the pattern of the SC is similar of the pattern of the CSD. We assume that, due to this sensitization effect for the SC, we would have found similar results to Nagle and collaborators [38] if we had used 10 repetitions of the stimulation instead of three.

Also, OCs in our experiment were not only composite vocalizations of SCs and STs but also calls of atypical high frequency or other rare forms. The STs in our experiment were similar to the ‘agitation trills’ of Mulligan & Olsen [40]; these trills were supposed to reflect a motivational state [40]. In this study, females responded significantly less with STs and OCs for the heterospecific zebra finch control song but performances of male canaries' songs did not affect the occurrence of these two types of signals. Also, we observed a habituation effect with the repetition of the stimulation for the ST whereas such habituation effect was not observed for the OC. We suggest that ST may be used by female canaries to signal a social motivation rather than a sexual motivation.

Finally, we found that the different call types are uttered by females at significantly different times relative to the beginning of male song. First of all, FSTs were uttered significantly before all other call types; female canaries emitted FSTs quickly after the beginning of the male song. Consequently, FSTs mostly overlapped the male song during the tests. Hence, FSTs were a clear response to male song suggesting interactive communication between mates. Also, SCs were uttered significantly later than STs after the start of the song; SCs were always produced after the end of the song, whereas STs could be produced both during and after the song. We did not measure latencies of CSDs because the camera was placed outside the sound-proof chamber and, even if the song can be heard, it was not possible to measure accurately the latencies of the CSDs relative to the start of the song. Nonetheless, CSDs were initiated during the song.

Our findings clearly support the copulation solicitation hypothesis [23] for the FST: they indeed are uttered during male song, they are positively correlated to CSDs, and mostly the response patterns are very similar for the CSDs and the FSTs. Overall, our findings support that female canaries could use FSTs to signal their readiness to copulate as multimodal signalling is often use in sexual signalling [44]. Therefore, FST is a good candidate to be a complementary or alternative measure to assess female canaries' preferences. This relationship between visual and vocal copulation displays could also be interpreted in light of knowledge on the copulation behaviour of wild canaries, the ancestors of the domestic canaries: during the breeding season, male wild canaries perform song flights over the nest and copulate with their partner [45]. We can assume that female canaries maximize their chances to copulate with a male when performing a FST and a CSD at the same time. The male may be attracted by the FST of the female even if the male is not at close range to its female and does not see the CSD. Anyway, the FSTs of female canaries are used in the final stage of the courtship. These female copulation calls are rarely studied in birds (but see for instance Balsby & Dabelsteen [46] in whitethroats, *Sylvia communis*; Dabelsteen [47] in blackbird, *Turdus merula*; Marler [19] in chaffinch, *Fringilla coelebs*); our experiment is the first to experimentally show the copulation solicitation function of a female-specific call in a songbird.

Despite the immediate female vocal response to the song of the male and, by consequence, the overlapping of the two acoustic displays, this vocal interaction is unlikely a duet, as duets imply that elements of the acoustic displays have a high level of alternation [14]. This does not seem to be the case in our experiment (M.A. and P.S., this study, personal observation) but we did not record natural male–female vocal interactions and we cannot exclude that they interactively adjust their vocalizations.

Yet, FSTs were produced with a heterogenous sound pressure level as already mentioned by Mulligan & Olsen [40]. We also observed many variations in the pressure level between FSTs of females and within the FSTs of a female (M.A. and P.S., personal observation). The overall moderate to low sound pressure level of the FST makes them a probable private signal [47,48], and female copulation trills are generally reported to sound quieter than most of the other vocalizations [47]. Finally, little is known about the private signals exchanged just before copulation, but the quietness of the copulation trills may reduce nest detection by predators [49] or copulation disruption by rivals [47]. Indeed, male–female vocal interactions can be affected by the social environment since neighbouring males can eavesdrop on rivals' courtship interactions [50] or on rivals' female begging calls [51] to seek extra-pair copulations; females may also eavesdrop on male–female vocal interactions to direct their preferences [52]. Further studies are needed to understand the evolution of male–female vocal interactions within a communication network.

The role of female signals has been historically overlooked [5,8,13,53]. We here show the copulation solicitation function of the FST in the domestic canary and that female canaries use multimodal signals to solicit copulation. Further studies are needed to investigate non-exclusive functions of these FSTs, and especially to test if they incite males to display.

## Supplementary Material

There is only one file entitled ‘Data of Female canaries’ signals to male song', which contain the data used for statistical analyses.

## Data accessibility

The datasets supporting this article have been uploaded as part of the electronic supplementary material.
